# ﻿Extension of the leafhopper genus *Multinervis* (Hemiptera, Cicadellidae, Megophthalminae, Agalliini) from Northern to Central Vietnam, with the description of one new species

**DOI:** 10.3897/zookeys.1233.136519

**Published:** 2025-03-26

**Authors:** Linda Semeraro, Jérôme Constant, Thai-Hong Pham

**Affiliations:** 1 Royal Belgian Institute of Natural Sciences, O.D. Taxonomy and Phylogeny, Entomology, Vautier Street 29, B-1000 Brussels, Belgium Royal Belgian Institute of Natural Sciences, O.D. Taxonomy and Phylogeny, Entomology Brussels Belgium; 2 Mientrung Institute for Scientific Research, Vietnam National Museum of Nature, VAST, 321 Huynh Thuc Khang, Hue, Vietnam Mientrung Institute for Scientific Research, Vietnam National Museum of Nature Huynh Thuc Khang Vietnam; 3 Graduate School of Science and Technology, Vietnam Academy of Science and Technology, 18 Hoang Quoc Viet, Hanoi, Vietnam Graduate School of Science and Technology, Vietnam Academy of Science and Technology Hanoi Vietnam

**Keywords:** Auchenorrhyncha, biodiversity, leafhopper, Membracoidea, plant pathogen, plant virus, taxonomy

## Abstract

A new species of the genus *Multinervis* Li & Li, 2013, *Multinervisphongdienensis***sp. nov.**, is described from two locations in Central Vietnam, Thưa Thien-Hue Province: Bach Ma National Park and Phong Dien District. It represents a second species for the genus and for the fauna of Vietnam, where the type species, *M.guangxiensis* Li & Li, 2013, has also previously been recorded from Northern Vietnam. Illustrations, a differential diagnosis to distinguish the two *Multinervis* species and a distribution map are provided. *Multinervisphongdienensis***sp. nov.** is characterized by the absence of forewing crossveins in the claval region, the reduced subgenital plates being almost entirely fused, the absence of a subgenital angular projection and the unusual lateral flanges of the male connective.

## ﻿Introduction

The tribe Agalliini Kirkaldy, 1901 is one of four recognised in the subfamily Megophthalminae Kirkaldy, 1906, along with Evansiolini Linnavuori & DeLong, 1977, Adelungiini Baker, 1915, and Megophthalmini Kirkaldy, 1906 (Dietrich 2005). This speciose tribe comprises 57 genera and more than 641 species (Dmitriev et al. 2022 onwards) worldwide. Currently, only 4 species in 3 genera of this tribe are known from Vietnam: *Austroagallianitobei* (Matsumura, 1912), *Multinervisguanxiensis* Li & Li, 2013, *Sangeetafyanensis* Viraktamath, 2011 and *S.linnavuorii* Viraktamath, 2011 (Viraktamath 2011; Dietrich et al. 2020). As a comparison, 48 species in 16 genera are recorded from this tribe in China, mostly from the southern regions (Viraktamath 2012; Li and Li 2013; Zhang 2014; Li et al. 2015). Species of this tribe feed mostly on grasses and broad-leaved herbs and may also feed on leguminous crops (Viraktamath 2011; Viraktamath et al. 2012; Li and Li 2013), with a number of species reported as disease vectors of plant viruses, phytoplasmas and Rickettsia-like organisms (RLO) (Nielson 1979; Viraktamath 2011; Viraktamath et al. 2012; Wilson and Turner 2021). This includes species of *Aceratagallia* Kirkaldy, 1907 (*A.curvata* Oman, 1933, *A.longula* van Duzee, 1894 and *A.sanguinolenta* (Provancher, 1872)) which transmit Potato yellow dwarf virus (PYD), *Agalliaconstricta* van Duzee, 1894, which is a vector of PYD and phytoplasmas (Nielson 1968; Trivellone 2019), *Agalliopsisnovella* (Say, 1830) which is a vector of clover club leaf RLO (Nielson 1968) and *Austroagalliatorrida* Evans, 1935, known to be a vector of rugose leaf curl virus (Grylls 1954; Nielson 1968; Fletcher et al. 2017).

The currently monotypic genus *Multinervis* Li & Li, 2013, is represented by the species *M.guangxiensis* Li & Li, 2013, which was described from Guangxi, China (Li and Li 2013). More recently the species was reported for the first time from Vietnam, Cuc Phuong National Park, Ninh Binh Province by Dietrich et al. (2020).

Following a two-week field expedition to Central Vietnam to collect insects in Bach Ma National Park and Phong Dien District in 2023, a second species of this genus was discovered and is described and illustrated in this paper, with a distribution map of the two known species.

## ﻿Material and methods

The specimens were collected by sweeping the vegetation with an entomological net and aspirating into a jar. The specimens were euthanized with ethyl acetate and later card-mounted for study. Following the methods outlined in Fletcher (2009), the abdomen of the male specimens was removed to examine the genitalia. The abdomens were first macerated in a 10% solution of potassium hydroxide (KOH), soaked at room temperature overnight (for at least 12 hours) and then thoroughly rinsed in 70% ethanol before transferring to glycerol in genitalia vials.

Specimens were examined and photographed under a Leica EZ4W stereomicroscope with an integrated camera. The photographs were stacked with CombineZP software and optimized with Adobe Photoshop CS3. Photographs of the habitat were taken with an Olympus Tough TG-6 camera.

The distribution map was produced with SimpleMappr (Shorthouse 2010).

Terminology of the general leafhopper morphology, wing venation and leg chaetotaxy follows Dietrich et al. (2022); for the male genitalia, it follows Dietrich et al. (2022) in part, and Blocker and Triplehorn (1985).

The following acronyms are used:

**CCRR** Centre for Conservation of Vietnam Natural Resources and Rescue of Animals and Plants, Phong Dien, Thưa Thien-Hue Province, Vietnam

**GUGC** Guizhou University, Guiyang Guizhou, China

**RBINS** Royal Belgian Institute of Natural Sciences, Brussels, Belgium

**VNMN** Vietnam National Museum of Nature, Hanoi, Vietnam

## ﻿Taxonomy

### ﻿Class Insecta Linnaeus, 1758

**Order Hemiptera Linnaeus, 1758**

**Suborder Auchenorrhyncha Duméril, 1806**

**Infra-order Cicadomorpha Evans, 1946**

**Superfamily Membracoidea Rafinesque, 1815**

**Family Cicadellidae Latreille, 1825**

**Subfamily Megophthalminae Kirkaldy, 1906**

**Tribe Agalliini Kirkaldy, 1901**

#### ﻿Genus Multinervis

Taxon classificationAnimaliaHemipteraCicadellidae

Li & Li, 2013

B508EC84-78B4-5D53-BC68-E103B24A8BD3

Multinervis Li & Li, 2013: 296 [description of the new genus Multinervis based on the type species, M.guangxiensis]; Dietrich et al. 2020: 265 [first record of the genus and species for Vietnam].

##### Type species.

*Multinervisguangxiensis* Li & Li, 2013.

##### Diagnosis.

The genus is recognised by a combination of characters including, a robust body; coloration generally brown; forewing, somewhat coriaceous, brown with contrasting pale yellow veins; crown very narrowly visible in dorsal view; face with striations along upper margin, dorsad of ocelli; ocelli in slight depressions, almost equidistant from each other as to compound eyes; granulose texture of head, pronotum, mesonotum and forewings; pronotum only weakly pitted, in lateral view slightly convex, anterior margin may be depressed, concave in lateral view; forewing bearing numerous accessory crossveins (veins appearing reticulated), with or without crossveins in claval region (between anal veins); forefemur with pale yellow and black/ dark brown bands; hindfemur with macrosetal formula 2+1; hindtibia with 7 macrosetae on AD margin. In male genitalia structures, the subgenital plates are fused at base; in lateral view, style apophyses are distinctly spiralled. Connective elongate, broader anteriorly than posteriorly (longer than broad), in ventral view, approximately racquet-shaped.

##### Differential diagnosis.

According to Li and Li (2013), *Multinervis* is most similar to three other genera of Agalliini – *Dryodurgades* Zachvatkin, 1946, *Paulagallia* Viraktamath, 2011 and *Sangeeta* Viraktamath, 2011. However, *Multinervis* differs from *Dryodurgades* in having a shorter aedeagal shaft without apical and subapical processes, an elongate connective, longer than wide and subgenital plates fused together in basal portion (in *Dryodurgades*, the aedeagal shaft is longer with branched apical and often subapical processes, the connective is broader than long and the subgenital plates are not fused basally). *Multinervis* differs from *Paulagallia* in having pronotum only weakly pitted, pygofer dorsal margin not deeply excavated, only slightly concave, without short stout setae apically, subgenital plates with no macrosetae, aedeagus without lateral ridges or apical processes, and with dorsal apodeme about half as long as aedeagal shaft, and connective elongate (in *Paulagallia*, the pronotum is coarsely pitted, the male pygofer has a deep, angular dorsal marginal excavation around midlength, apex of pygofer bears short stout setae, subgenital plates bear macrosetae, the aedeagal shaft may have lateral ridges and small tooth-like processes apically, the aedeagal dorsal apodeme is almost as long as the shaft and the connective is about as long as wide). Based on Viraktamath (2011), a further characteristic by which *Multinervis* can be distinguished from the two above-mentioned genera includes the ocelli being approximately equally distant from each other as they are to the compound eyes (in *Dryodurgades* and *Paulagallia* the ocelli are closer to compound eyes than to each other). Li and Li (2013) considered another genus, *Sangeeta* Viraktamath, 2011, to be close to *Multinervis* based on the striations of the face, but the latter differs in that it has multiple accessory forewing crossveins (while *Sangeeta* does not have additional crossveins). In *Multinervis* the face width across the eyes is greater than the face length (in *Sangeeta* the face is longer than wide), the number of AD macrosetae on the hindtibia is 7 (*Sangeeta* has 6±1 macrosetae), the dorsal margin of the pygofer is slightly concave (while in *Sangeeta* the pygofer has a distinct almost right-angled excavation) and the pygofer apex and subgenital plates are without macrosetae (in *Sangeeta* the pygofer apex has some stout setae and subgenital plates have a row of macrosetae).

##### Distribution.

(Fig. 6) China (southern) and Vietnam (Northern and Central).

##### Hosts.

Unknown.

##### Species list.

(type locality indicated by *):

*Multinervisguangxiensis* Li & Li, 2013 [China, Guangxi Province* • Vietnam, Ninh Binh Province]. Holotype: GUGC.

*Multinervisphongdienensis* sp. nov. [VIETNAM, Thưa Thiȇn-Hué Province*]. Holotype: VNMN.

#### ﻿ Multinervis phongdienensis sp. nov.

Taxon classificationAnimaliaHemipteraCicadellidae

E5F42735-8A78-5A7A-8274-1CD7B2E87B1E

https://zoobank.org/8062B470-7E73-497C-AC0D-AA72EC3F0685

**Figure 1.**
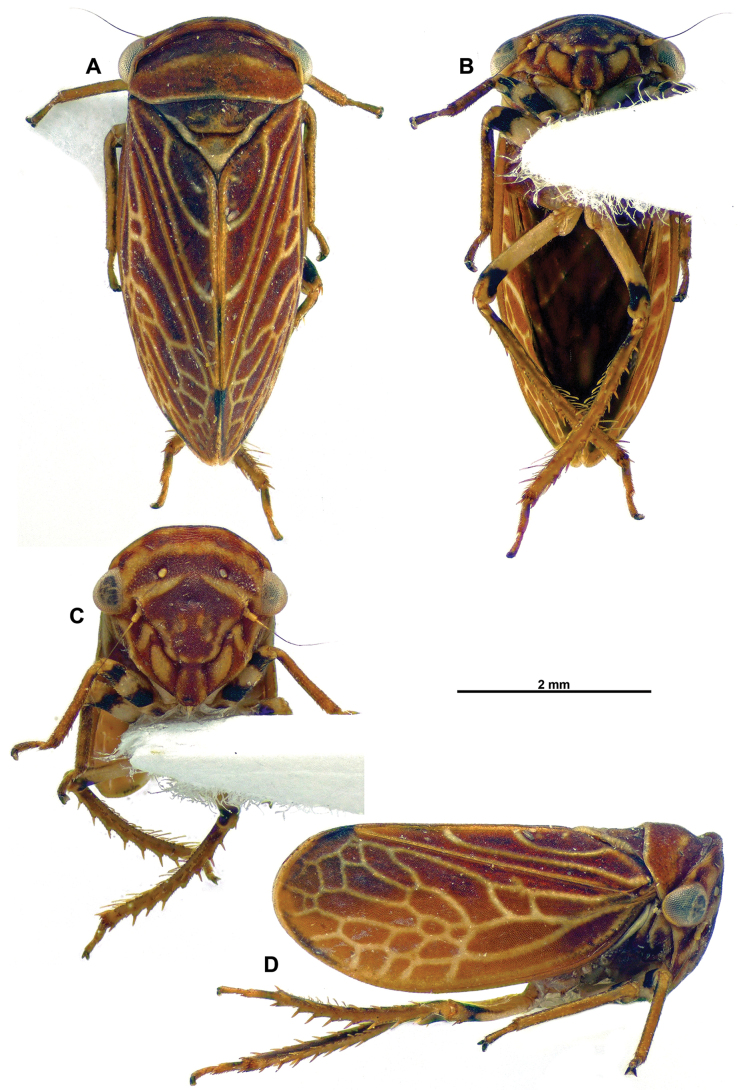
*Multinervisphongdienensis* sp. nov., holotype ♂ (VNMN) **A** dorsal habitus **B** ventral habitus **C** face, perpendicular view **D** lateral habitus.

**Figure 2.**
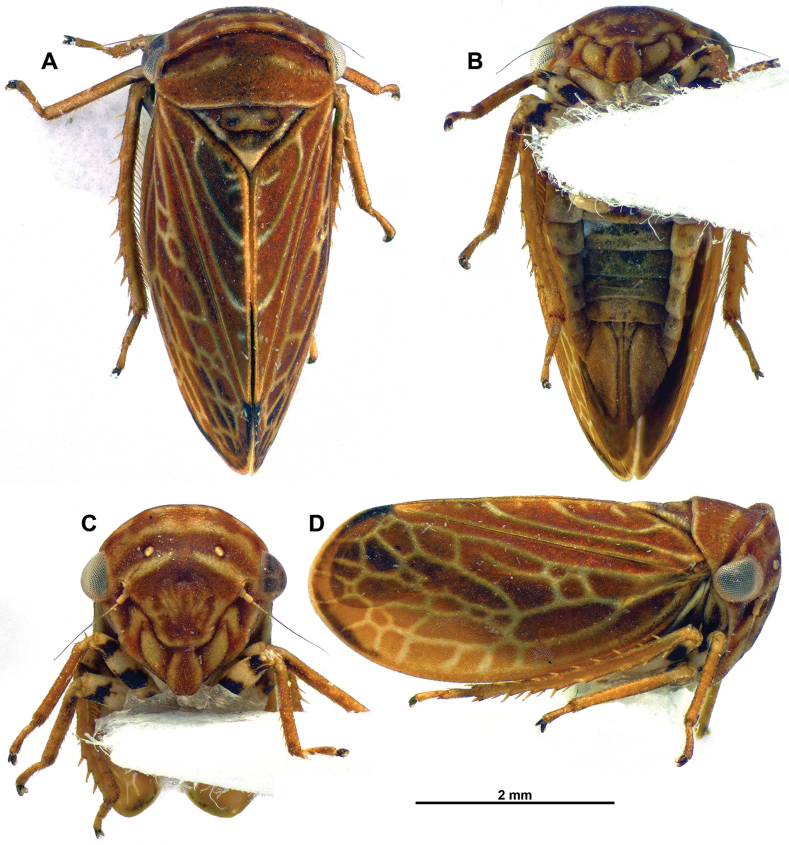
*Multinervisphongdienensis* sp. nov., paratype ♀ (VNMN) **A** dorsal habitus **B** ventral habitus **C** face, perpendicular view **D** lateral habitus.

**Figure 3.**
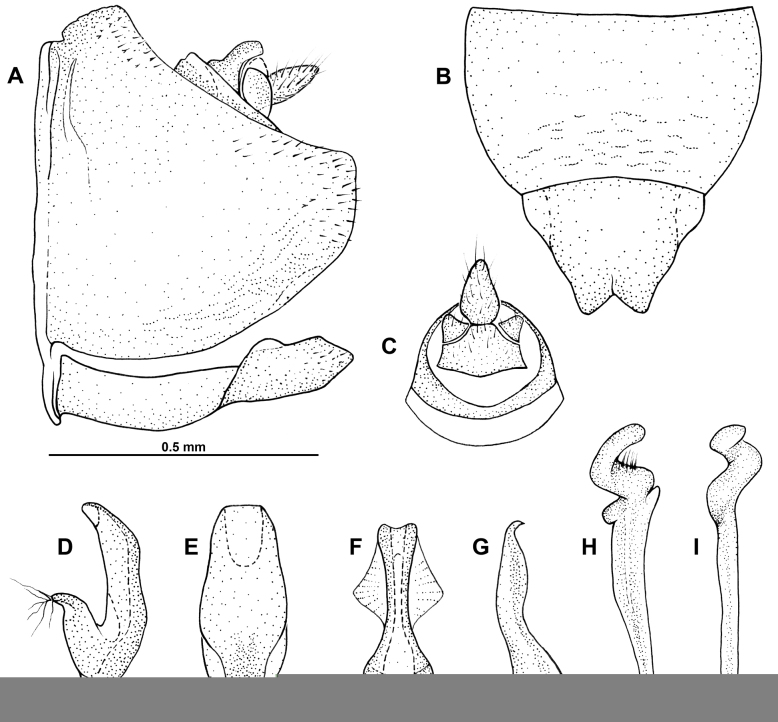
*Multinervisphongdienensis* sp. nov., holotype ♂ (VNMN) **A** pygofer, subgenital plate and pregenital sternite, left lateral view **B** pregenital sternite and subgenital plates, ventral view **C** anal tube, dorsal view **D** aedeagus, left lateral view **E** aedeagus, caudal view **F** connective, ventral view **G** connective, left lateral view **H** left style, dorsal view **I** left style, lateral view.

**Figure 4.**
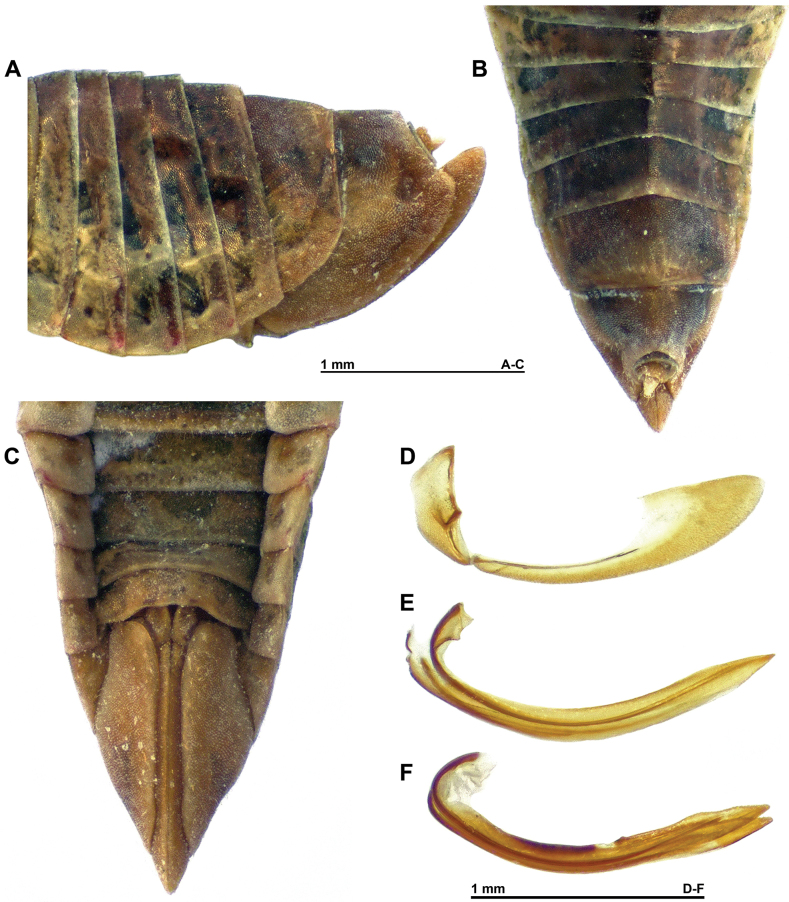
*Multinervisphongdienensis* sp. nov., paratype ♀ (VNMN) **A** abdominal tergites and pygofer, left lateral view **B** abdominal tergites and pygofer, dorsal view **C** abdominal sternites, pygofer and ovipositor, ventral view **D** second valvifer and third valvula, lateral view **E** first valvula, lateral view **F** second valvulae, lateral view.

**Figure 5.**
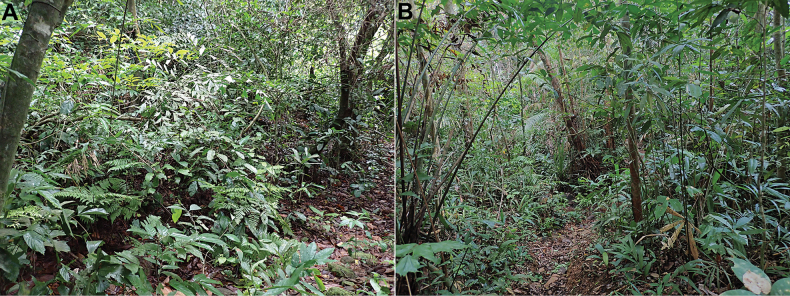
*Multinervisphongdienensis* sp. nov., habitat **A** Bach Ma National Park, low altitude, 15 May 2023 **B** Phong Dien District, 23 May 2023.

**Figure 6.**
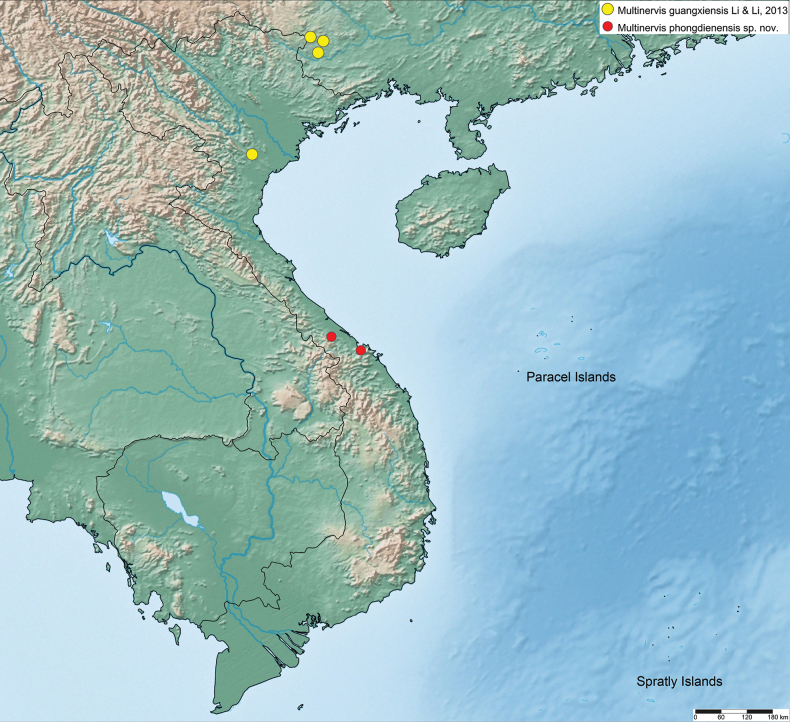
Distribution map of *Multinervisguangxiensis* Li & Li, 2013 and *M.phongdienensis* sp. nov.

Figs 1 , 2 , 3 , 4 , 5 , 6

##### Type material.

***Holotype*** ♂ (Figs 1, 3), Vietnam • Thưa Thiȇn-Hué Province, Bach Ma National Park; low altitude; 16°13'14"N, 107°53'10"E; 17 May 2023; alt. 100–200m; J. Constant and L. Semeraro leg.; I.G. 34.640; VNMN. ***Paratypes***, Vietnam • 1♀; same data as in holotype; RBINS • 1♀; same data as in holotype; VNMN • 1♂; Thưa Thiȇn-Hué Province, Phong Dien District; 16°30'27"N, 107°16'05"E; 23 May 2023; alt. 350–400m; J. Constant and L. Semeraro, leg.; I.G. 34.640; RBINS.

##### Diagnosis.

Body robust. Colouration chestnut brown contrasted with pale yellow veins and markings on face. Forewing with veins loosely reticulated mainly on apical half, crossveins in claval area absent; anal vein A1 strongly curved from base. Subgenital plates relatively short, only just reaching posterior margin of pygofer (see Fig. 3A), subgenital plates fused along almost entire length. Aedeagal shaft, narrow in lateral view. Connective elongate, racquet-shaped, broader anteriorly than posteriorly, and in caudal view, posterior shaft with lateral membranous triangular flanges. Female seventh sternite much wider than long.

##### Differential diagnosis.

*Multinervisphongdienensis* sp. nov. can be differentiated from *M.guangxiensis* in body length, being slightly smaller (4.5 mm versus 4.8–5.2 mm in *M.guangxiensis*); the general body shape being more squat (around 2.4× longer than wide at widest point versus 2.75×); the frontoclypeus mostly patchy chestnut brown medially (face generally paler and with a large pale yellow patch medially in *M.guangxiensis*); forewing venation is less reticulated (fewer crossveins), crossveins between anal veins are absent and there are fewer crossveins between discal and costal cells (versus crossveins present between the anal veins and relatively densely reticulated venation between discal and costal cells) anal vein A1 is strongly curved from near the base, not parallel with anal vein Pcu (versus very slightly curved, more or less parallel with Pcu); in the male genitalia, the subgenitial plates appear shorter and only just reaching posterior margin of the pygofer (Fig. 3A) (versus subgenital plates exceeding the posterior margin of the pygofer); in lateral view, the subgenital plate has a slightly rounded projection basodorsally, digitate projection absent (versus distinct digitate projection near base); in ventral view, the subgenital plates appear short and fused for most of their length (versus subgenital plates more elongate, only fused along basal 1/3); the aedeagus is similar in shape to *M.guangxiensis* but is narrower in lateral view; female seventh sternite posterior margin mostly transverse to very weakly broadly concave (versus posterior margin with a deep medial u-shaped notch).

##### Description.

***Measurements and ratios***. Body length, ♂ holotype, 4.5 mm; paratypes ♂, 4.5 mm; 2 ♀♀, 4.5mm. Proportion of body length 2.4× the width (measured across widest part of body). Head across eyes slightly wider than pronotum. Pronotum 2.5× as broad as long. Scutellum 1.25× length of pronotum along midline.

***General body colouration*.** Chestnut brown with contrasting pale-yellow markings and tegminal venation. Colouration of males and females mostly identical.

***Head*** (Figs 1A–D, 2A–D) Head very short, visible in dorsal view, crown slightly produced dorsad with upturned lip-like margin, slightly emarginate posterior to compound eyes; crown, brown with diffuse paler brown patches; face, striated across dorsal margin, slightly rugose around ocelli, each ocellus in slight depression; colour of face mostly chestnut brown with distinctive pale yellow pattern – brown and yellow bands across dorsal area of face, brown band across ocelli, ventral to ocelli, pair of pale yellow bands tapered to a point mesally; frontoclypeus and anteclypeus chestnut brown with patches of pale yellow; lora pale yellow, brown along sutural margins of anteclypeus and lora; genae brown with pale yellow longitudinal band medially.

***Thorax*** (Figs 1A, 2A) Pronotum with fine, shallow pitting posteriorly, depressed along anterior margin, posterior margin transverse or only weakly concave; pronotal colour generally brown – darker brown band across anterior margin, pair of diffuse pale-yellow stripes posterior to depression, medial disc chestnut brown, posterior margin bordered with very fine pale yellow outline. Proepisternum not visible. Mesonotum with paired shallow oval depressions medially, scutellar suture transverse, recurved laterally; mesonotum and scutellum brown anteriorly, pale brown/ yellow medially, pale yellow apically, scutellar suture brown.

***Forewings*** (Figs 1A, D, 2A, D) Generally opaque chestnut brown, surface granular, apical margin outlined dark brown, from apex of claval suture to costal margin; veins pale yellow; basal half of costal cells with few crossveins, clavus and brachial cells without complete crossveins, female specimens with additional pale yellow flecks stemming from anal margin; anal vein A1 strongly curved at base, not parallel with vein Pcu; apical half of forewing, with numerous crossveins, appearing loosely reticulate.

***Legs*** (Figs 1B−D, 2B−D) Pale brown/ yellow; tibiae pale brown/ pale yellow; fore femora striped with black and pale yellow bands; mid femora mostly pale yellow with black band preapically; hind femora pale yellow with longitudinal black markings dorsally, black band apically. Hind femoral macrosetae 2+1; number of macrosetae on hind tibia AD row = 7.

***Abdomen*** In males and females, tergites generally brownish, paler brown medially, darker brown in patches, laterally pale yellowish; sternites pale brown to pale yellowish.

***Terminalia*** ♂ (Fig. 3) Pygofer, broad basally, dorsal margin weakly concave, posterior margin subquadrate, ventral margin broadly convex; short fine setae on posterior 1/8 of pygofer. Anal tube short, not reaching posterior margin of pygofer. Subgenital plates, only just reaching posterior pygofer margin; in lateral view, plates with laterobasal membranous lobe directed dorsad; in ventral view, plates fused along almost entire length; plates tapering apically, produced as triangular points, or slightly rounded at apex, posterior margin medially forming distinct v-shaped notch. Aedeagus symmetrical; more than half height of pygofer, positioned almost horizontally, apex directed anterodorsad, reaching near base of anal tube; gonopore positioned apically on ventral margin; in lateral view, shaft narrow, slightly sinuate along posterior margin; dorsal apodeme less than half length of shaft; well-developed preatrium; in caudal view, aedeagus broad, lateral margins slightly convex, narrowing slightly towards apex. Connective, elongate, longer than wide, sclerotised portion wider anteriorly than posteriorly; posterior portion articulated with preatrium of aedeagus; in lateral view, oriented obliquely within pygofer, sinuous in profile; in ventral view, anterior portion, approximately racquet-shaped, arms divergent and then convergent distally; sclerotised arms separated throughout, medially membranous, anterior portion of arms enclosed by membranous tissue; elongate posterior stem of connective with membranous lateral triangular flanges. Style basal part elongate; apex of style, reaching near posterior pygofer margin; apophyses spiraled, with fine preapical setae visible from dorsal aspect.

***Terminalia*** ♀ (Fig. 4) Seventh sternite pale brownish/ yellow, wider than long (length medially around 1/5 its width); transverse, posterior margin straight to broadly, weakly concave, lateral posterior angles slightly rounded. Pygofer pale yellow with some diffuse brown patches dorsally, surface slightly granulate; ovipositor pale yellow; apex of ovipositor slightly but distinctly exceeding length of pygofer; valvulae similar in form to generic description (Li and Li 2013); first valvulae with reticulated area along dorsoapical portion of shaft; second valvulae with small tooth-like process posterior to medial dorsal hyaline area, small teeth along dorsoapical margin.

##### Etymology.

The species epithet is derived from the collection locality, Phong Dien District which includes the site of CCRR, one of the two locations in Thưa Thiȇn-Hué Province, in which this species was found.

##### Biology and habitat.

(Fig. 5) The biology of this species is not known. Specimens were collected in subtropical evergreen rainforest in a region bordering the Northern and Southern Vietnam lowland rainforests and Southern Annamites montane forests. In Bach Ma, specimens were only collected by sweeping the vegetation at low altitude (100–200 m) along a track. No specimens were collected at the higher cooler climate altitudes (towards the summit). However, in Phong Dien one specimen was collected at 350–400 m.

##### Host.

Unknown.

##### Distribution.

(Fig. 6) Vietnam • Thưa Thiȇn-Hué Province, Bach Ma National Park and Phong Dien District.

##### Comments.

The holotype of the type species of *Multinervis* (*M.guangxiensis*) was not examined in this study, but specimens were compared to the description of the genus and species and the photographs and illustrations as presented in Li and Li (2013: figs 1–24).

## ﻿Discussion

Following the description of this new species, there are now five species of Agalliini recorded from Vietnam and the total number of *Multinervis* species has increased to two, with both species recorded from Vietnam.

*Multinervis* was recorded for the first time in Vietnam by Dietrich et al. (2020). The specimen of *M.guangxiensis* photographed in Dietrich et al. (2020: fig. 21X) appears to have more densely reticulated veins on the forewing compared to the original description of this species in Li and Li (2013, figs 1–3). This may indicate intraspecific crossvein variability within *Multinervis*. In *M.phongdienensis* sp. nov., however, the crossvein pattern varies only slightly between specimens and is generally consistent between all four specimens examined. The male genitalia structures remain the most reliable feature for species determination.

The diagnostic characters of *Multinervis* are slightly redefined in this study to capture the additional range of character states, as found in the new species. For example, the striations on the face above the ocelli are described in Li and Li (2013) as being conspicuous in this genus but are only faintly visible along the apical margin of the face of *M.phongdienensis* sp. nov. In the male genitalia, the subgenital plates are originally described as being elongate and only fused in the basal 1/3, while in the new species, the subgenital plates are relatively short and fused for almost the entire length of the plate. The dorsally directed digitate process on the basal half of the subgenital plate was described as a character of *Multinervis*, but it is absent in the new species, possibly replaced by a membranous lobe.

*Multinervis* species have an elongate connective, which is approximately racquet-shaped. While this elongate connective feature is shared with a few other Agalliini genera, such as *Brasa* Oman, 1936 and *Humpatagallia* Linnavuori & Viraktamath, 1973 (Linnavuori and Viraktamath 1973; Nielson and Freytag 1998), it differs from many other genera in the tribe, which have a relatively wide and short connective. The connective shape and the presence of tegminal crossveins represent relatively rare features in the Old World Agalliini (Viraktamath 2011).

Additionally, the connective of *M.phongdienensis* sp. nov. is particularly unusual in having membranous flanges on the lateral margins of the posterior stem. The flanges do not appear to be present in *M.guangxiensis* based on the descriptions and illustrations in Li and Li (2013). It is considered a peculiar diagnostic feature of *M.phongdienensis* sp. nov.

The new species described in this study only represents one of many species of Cicadellidae to be described from the 2023 expedition to Bach Ma National Park and Phong Dien District (including the Phong Dien Nature Reserve Nature Reserve and CCRR). It is complimentary to the work by Constant et al. (2023, 2024) and Constant and Pham (2024) in which one new genus and 11 new species of planthoppers (Nogodinidae, Tropiduchidae and Issidae, respectively) were described from specimens collected during the same expedition, indicating a rich biodiversity of Auchenorrhyncha in the Thưa Thien-Hue Province region.

## Supplementary Material

XML Treatment for Multinervis

XML Treatment for Multinervis phongdienensis

## ﻿Additional information

### Author ORCIDs

Linda Semeraro https://orcid.org/0000-0002-4057-4733

Jérôme Constant https://orcid.org/0000-0003-0254-0863

Thai-Hong Pham https://orcid.org/0000-0002-4763-3679

### Data availability

All of the data that support the findings of this study are available in the main text.
